# Prokaryotic expression of chimeric GFP-hFc protein as a potential immune-based tool

**DOI:** 10.22099/mbrc.2021.39728.1588

**Published:** 2021-09

**Authors:** Thanh-Tan Nguyen, Hai-Vy Vo-Nguyen, Hieu Tran-Van

**Affiliations:** 1 Department of Molecular and Environmental Biotechnology, Faculty of Biology and Biotechnology, University of Science, Ho Chi Minh City, Vietnam; 2 Vietnam National University, Ho Chi Minh City, Vietnam

**Keywords:** GFP, hFc-tag, GPF-hFc, protein A/G-coated magnetic beads

## Abstract

GFP is an old-yet-powerful protein marker, which has been widely used in molecular biotechnology due to its capacity of exhibiting bright green fluorescence when exposed to ultraviolet light. The hFc region of IgG antibodies is a specific binding ligand of expressed receptors on immune cells with well-known cellular-associated functions like opsonization and phagocytosis. In this present study, we proceeded to fuse *gfp-hfc* gene into pET-28a to create a recombinant pET-28a-gfp-hfc vector. The expression of GPF-hFc was induced by IPTG and confirmed using SDS-PAGE and followed by Western blot probed with 6xHis antibodies. This chimeric protein was utilized in specific binding experiments with protein A/G-coated magnetic beads using a fluorescence microscope. Due to its fluorescence and binding ability, GFP-hFc could be used as a model molecule for monitoring molecule detection studies, tracking nanoparticle migration and distribution, or stimulating immune responses.

## INTRODUCTION

Along with the development of modern biotechnology, the discovery of marker proteins is one of the leading strategies in the field of molecular biology. Green Fluorescent Protein (GFP) derived from jellyfish *Aequorea victoria *can absorb ultraviolet to blue light and emit green fluorescence, which possesses many benefits. It has been utilized as a marker to study gene expression, and protein trafficking, targeting, location and interactions. Moreover, GFP is not toxic to most types of cells, and can be recognized without harming the cell [1, 2]. In addition, GFP is resistant to heat, detergents, alkaline pH, and high salt concentration. Thus, GFP has been widely used in transgenic monitoring systems and promoter activity evaluation of gene expression. From the potentials above, we conducted to fuse hFc-tag of IgG with GFP to form chimeric protein GFP-hFc expressed in *Escherichia coli* with many outstanding advantages [3]. From the perspective of immunology, the hFc-tag is a part of the antibody structure which facilitates the interaction with immune cells and activates immune reactions of macrophages, complements, and opsonization [4]. Besides, hFc-tag also specifically binds to protein A/G as an application for the detection of protein A/G-coated magnetic beads to separate pathogens, target and deliver drugs to organs. Therefore, GFP-hFc could be deployed as a recombinant protein model in detecting and tracking target of interest, rerouting magnetic beads, and stimulating immune responses [4].

## MATERIALS AND METHODS

**Construction of the expression vector pET28a-gfp-hfc:** *hfc* gene was amplified from *plg-mIL1RAcPex* plasmid by PCR with specific primers 5’*BamH*I-3’*Xho*I, resulting in a 751 bp product. The pET28a-gfp plasmid was digested with *BamH*I and *Xho*I (Thermo Scientific). PCR product and digested plasmid were incubated with the ratio of 1:1 at 37^o^C in 30 minutes. Then the mixture was transformed into competent *E. coli* DH5α. The transformants were initially screened on a kanamycin-containing LB agar plate, then re-screened by PCR with specific primers and 5’*BamH*I-T7ter. Finally, the verified pET28a-gfp-hfc plasmid was transformed into *E. coli* BL21(DE3) cells for protein expression.

**Expression and confirmation of recombinant GFP-hFc protein in** ***E. coli*** **BL21 (DE3): **The expression of recombinant protein was conducted as described with some modifications [5-7]. The pET28a-gfp-hfc obtained from previous steps was transformed into competent *E. coli* BL21 (DE3). Positive colonies were inoculated in LB media shaking tubes supplemented with kanamycin and allowed to grow at 37^o^C overnight. The cultures were sub-cultured at 1:10 (v/v) and inoculated at 37^o^C until optical density (OD_600 nm_) reached 0.8-1.0. At this point, isopropyl-beta-thio-galactopyranoside (IPTG) was added to a final concentration of 0.5 mM and protein expressions were performed with 200 rpm at 16^o^C in 16 hours. Recombinant protein expression was confirmed by SDS-PAGE and Coomassie Brilliant Blue stained, followed by Western blot. The proteins were blotted onto a nitrocellulose membrane and were detected by 6xHis-antibody-HRP (1:20,000) (ProteinTech). 

**Binding evaluation of recombinant GFP-hFc with protein A-coated magnetic beads: **The induced *E. coli* BL21 (DE3)/pET28a-gfd-hfc cells were sonicated to obtained supernatant-containing GFP-hFc, which was used to interact with prepared protein A/G-coated magnetic beads (Thermo Scientific™ Pierce™ magnetic beads). Non-purified GFP-hFc was rotatedly incubated with magnetic beads in 45 minutes at room temperature. Then, the mixture was washed with PBS (pH=7.4) three times, and protein A/G-coated magnetic beads were collected and mounted for fluorescence microscope screening. Non-purified GFP was utilized as a negative control.

## RESULTS AND DISCUSSION

**Figure 1:**
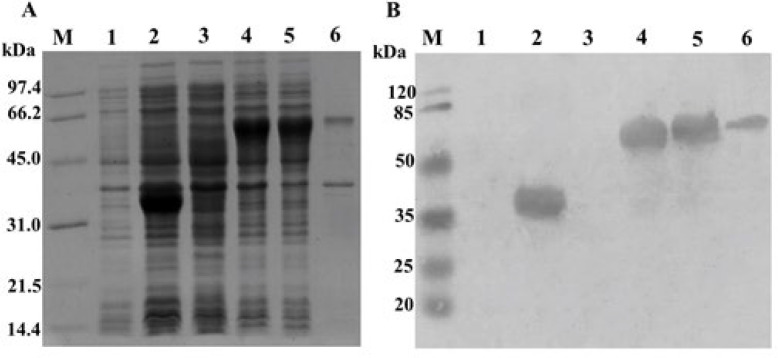
Coomassie brilliant staining of expressed GFP-hFc analyzed by SDS-PAGE on 17.5% gel (A) and confirmed by Western blot probed with anti-His-tag (B) M, protein maker (A) and pre-stained protein marker (B); 1, *E.coli *BL21 (DE3) (+IPTG); 2, *E.coli* BL21 (DE3)/pET28a-gfp (+IPTG); 3, *E.coli* BL21 (DE3)/pET28a-gfp-hfc (-IPTG); 4-6, *E.coli* BL21 (DE3)/pET28a-gfp-hfc (+IPTG); 4, total phase; 5, soluble phase; 6, insoluble phase

The *hfc* gene was successfully cloned into the expression vector pET28a-gfp to construct the pET28a-gfp-hfc vector. The recombinant GFP-hFc protein was induced for expression from *E.coli* BL21 (DE3)/pET28a-gfp-hfc with a molecular mass approximately of about 50 kDa, confirmed by SDS-PAGE analysis and Western blot. The results in Figure 1 showed over-expressing protein bands in lane 5 with the size at about 50 kDa equal to the predicted size. There was no over-expression band in the negative control.

**Figure 2:**
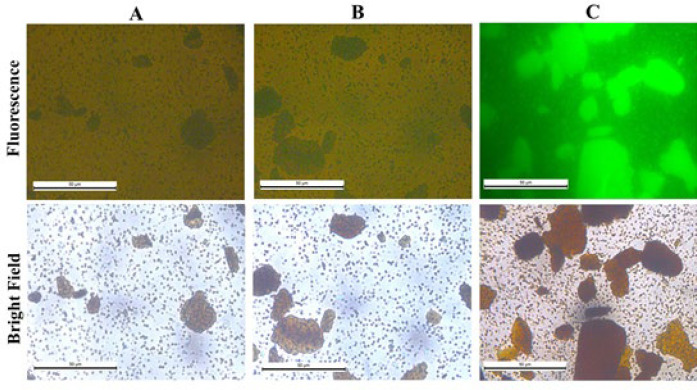
Detection of GFP-hFc interaction with protein A/G-coated magetic beads by fluorescence microscope. A, PBS; B, GFP; C, GFP-hFc

In addition, recombinant GFP-hFc protein was designed to fuse with the 6xHis-tag at the C-terminal, so the presence of recombinant GFP-hFc was confirmed by 6xHis-antibody-HRP in Western blot. The results indicated that the protein excessively expressed in the SDS-PAGE gels was recombinant GFP-hFc protein and this protein was expressed mostly in the soluble phase (Fig. 1, lane 5). Thus, the recombinant GFP-hFc was successfully expressed. In comparison with Figure 2A and Figure 2B, GFP-hFc showed significantly stronger fluorescent signals in Figure 2C. These results indicated that GFP-hFc had better binding to protein A/G-coated magnetic beads than GFP alone, since the hFc-tag acted as an effective linker. 

In this study, we designed, cloned, and expressed a chimeric recombinant protein GFP fused with the hFc-tag of IgG antibodies and also demonstrated a specific binding experiment via protein A/G-coated magnetic beads. GFP has been widely used as a model protein due to its fluorescent signal, which provides the most visualized and certain results that could be easily achieved. In further experiments, GFP-hFc fused with another protein can be purified using protein A/G-coated magnetic beads and the purification efficiency can simply be screened by a fluorescence microscope. This could help shorten and simplify the needed steps in a traditional protein purification protocol. 

The pET28a-gfp-hfc vector was successfully generated. To our knowledge, we documented for the first time a prokaryotic-derived heterologous protein of GFP-fused hFc in order to produce a potential immune-based tool. Moreover, the recombinant protein of interest GFP-hFc was expressed predominantly in the soluble fraction using *E. coli* BL21 (DE3) system [8] without periplasmic expression located [9] or any refolding step required [10]. Western blot with the anti-6xHis-HRP antibody of the recombinant protein showed a molecular weight of about 50 kDa was consistent with the predicted size. The protein exhibited the biological properties of fluorescence blue emission when observed under a fluorescence microscope and the specific association of fusion hFc-tag with protein A/G-coated magnetic beads.

Furthermore, the following stage of our research will be protein purification and confirmation of the protein immunogenicity [2, 4]. Subsequently, recombinant protein GFP-hFc is expected to act as a model tool for magnetic beads research, targeting and inducing immune responses *in vivo *or *in vitro *[2].
